# Discovery and surveillance of viruses from salmon in British Columbia using viral immune-response biomarkers, metatranscriptomics, and high-throughput RT-PCR

**DOI:** 10.1093/ve/veaa069

**Published:** 2020-09-01

**Authors:** Gideon J Mordecai, Emiliano Di Cicco, Oliver P Günther, Angela D Schulze, Karia H Kaukinen, Shaorong Li, Amy Tabata, Tobi J Ming, Hugh W Ferguson, Curtis A Suttle, Kristina M Miller

**Affiliations:** 1 Department of Medicine, University of British Columbia, 2775 Laurel Street, 10th Floor Vancouver, BC Canada V5Z 1M9, Canada; 2 Pacific Biological Station, Fisheries and Oceans Canada, 3190 Hammond Bay Rd, Nanaimo, BC V9T 6N7, Canada; 3 Pacific Salmon Foundation, 1682 W 7th Ave, Vancouver, BC V6J 4S6, Canada; 4 Günther Analytics, 402-5775 Hampton Place, Vancouver, BC, V6T 2G6, Canada; 5 School of Veterinary Medicine, St George’s University, True Blue, GrenadaWest Indies; 6 Department of Earth, Ocean and Atmospheric Sciences, University of British Columbia, Vancouver, Canada; 7 Department of Microbiology and Immunology, University of British Columbia, 1365 - 2350 Health Sciences Mall Vancouver, British Columbia Canada V6T 1Z3; 8 Department of Botany, University of British Columbia, 3156-6270 University Blvd. Vancouver, BC Canada V6T 1Z4, Canada; 9 Institute for the Oceans and Fisheries, University of British Columbia, 2202 Main Mall, Vancouver, BC V6T 1Z4, Canada

**Keywords:** virus discovery, viral ecology, in situ hybridisation, metatranscriptomics, aquaculture, fisheries

## Abstract

The emergence of infectious agents poses a continual economic and environmental challenge to aquaculture production, yet the diversity, abundance, and epidemiology of aquatic viruses are poorly characterised. In this study, we applied salmon host transcriptional biomarkers to identify and select fish in a viral disease state, but only those that were negative for known viruses based on RT-PCR screening. These fish were selected for metatranscriptomic sequencing to discover potential viral pathogens of dead and dying farmed Atlantic (*Salmo salar*) and Chinook (*Oncorhynchus tshawytscha*) salmon in British Columbia (BC). We found that the application of the biomarker panel increased the probability of discovering viruses in aquaculture populations. We discovered two viruses that have not previously been characterised in Atlantic salmon farms in BC (Atlantic salmon calicivirus and Cutthroat trout virus-2), as well as partially sequenced three putative novel viruses. To determine the epidemiology of the newly discovered or emerging viruses, we conducted high-throughput reverse transcription polymerase chain reaction (RT-PCR) and screened over 9,000 farmed and wild salmon sampled over one decade. Atlantic salmon calicivirus and Cutthroat trout virus-2 were in more than half of the farmed Atlantic salmon we tested. Importantly we detected some of the viruses we first discovered in farmed Atlantic salmon in Chinook salmon, suggesting a broad host range. Finally, we applied *in situ* hybridisation to determine infection and found differing cell tropism for each virus tested. Our study demonstrates that continual discovery and surveillance of emerging viruses in these ecologically important salmon will be vital for management of both aquaculture and wild resources in the future.

## 1. Introduction

The global demand for seafood is rising, whilst many wild fisheries are decreasing in productivity (Worm and Branch 2012). There is extensive interest in the potential impact of infectious disease on wild salmon populations. The 2009 decline of Canada’s largest salmon fishery, Fraser River sockeye salmon (*Oncorhynchus nerka*), instigated a public enquiry into declining salmon stocks, in which infectious disease was highlighted as an area deserving of more study (Cohen 2002). Meanwhile, a recent federal report found that half of Canada’s Chinook salmon (*Oncorhynchus tshawytscha*) populations are endangered, with nearly all other populations in decline (Environment and Climate Change Canada 2018). Aquaculture production is growing rapidly to meet the increased seafood demand, and its expansion provides increased opportunity for the transmission of emerging viruses and the evolution of virulence (Kennedy et al. 2016; Kibenge 2019). Disease can be a major factor limiting aquaculture production (Zhang and Gui 2015) and lead to substantial economic loss (Walker and Winton 2010; Lafferty et al. 2015). Although there is a growing body of research on the risk posed by pathogens and parasites associated with aquaculture on wild marine species (Godwin et al. 2015, 2018; Groner et al. 2016; Teffer et al. 2018; Bass et al. 2019), there has been little research that considers the risk of emerging viruses in this context. In fact, a recent Canadian government audit criticised government regulatory bodies for not addressing this knowledge gap and the potential risk to wild salmon from emerging viruses associated with the aquaculture industry (Spring Reports of the Commissioner of the Environment and Sustainable Development to the Parliament of Canada 2018).

Factors affecting salmon populations are multi-faceted and complex, but accumulating evidence suggests that infectious disease may play a role in the collapse of wild salmon populations in the Eastern Pacific. A range of pathogens have been linked to mortality in adult wild Pacific salmon (Miller et al. 2014; Teffer et al. 2018; Bass et al. 2019), but there are few data on the role of infectious diseases in the >90 per cent mortality of migratory juvenile salmon in the ocean. Gene expression profiles consistent with an immune response to viruses have been associated with mortality in wild migratory smolts and adults (Miller et al. 2011; Jeffries et al. 2014), as well as in unexplained aquaculture mortalities of salmon in marine net pens in British Columbia (BC) (Miller et al. 2017; Di Cicco et al. 2018). Together, these suggest that there are a range of viruses that may contribute to decreased survival of migratory salmon in BC. Moreover, there are concerns about the impact of emerging infectious agents associated with the expansion of salmon enhancement hatcheries (Naish et al. 2007; Mordecai et al. 2019) and salmon aquaculture in BC, which operate in the same waters through which wild Pacific salmon migrate (Morton et al. 2017).

The number of documented RNA viruses is undergoing rapid growth, largely since the next generation sequencing and associated metatranscriptomic boom of the last decade (Bolduc et al. 2012; Shi et al. 2016, 2018; Debat 2018; Greninger 2018). As a result, we are gradually uncovering previously unknown viral diversity, known as viral dark matter (Krishnamurthy and Wang 2017; Obbard et al. 2020). However, metagenomics has limitations, and there are barriers to viral discovery—screening a large number of samples is costly and time consuming and viral genomes are often outnumbered by orders of magnitude amongst the host, bacteria, and other contaminants (Daly et al. 2015; Rosani et al. 2019; Zhang et al. 2019). Furthermore, huge amounts of data are generated in a ‘sequence and see’ approach to metagenomics, which can be costly and time consuming to analyse, and there is no guarantee that the genomes that are found are biologically relevant, or even infect the host, which was sampled. Our knowledge of viruses in non-mammalian vertebrates is comparatively scarce, but growing rapidly, and most families of RNA viruses once thought to be restricted to mammals are now known to infect or be associated with fish metatranscriptomes, some of which appear to be ancient relatives of important human pathogens (Shi et al. 2018; Zhang et al. 2018; Mordecai et al. 2019).

Studying disease in wild populations is exceedingly complex (Rhyan and Spraker 2010), and although terrestrial strategies can include treating for known infectious agents, fish health investigations often begin with discovery. In the ocean, mortality events are rarely observed; sampling efforts solely capture live fish, and weak and dying fish are probably predated before the disease progresses to mortality (Miller et al. 2014, 2017). In order to surmount some of these difficulties, we applied a host viral disease development (VDD) biomarker panel shown to be predictive of an active RNA virus infection in salmon (Miller et al. 2017; Di Cicco et al. 2018) to select fish for metatranscriptomic sequencing, followed by molecular surveillance and *in situ* hybridisation (ISH) to prove infection.

## 2. Materials and methods

### 2.1 Sample collection

We applied similar sequencing and screening approaches to those used previously (Mordecai et al. 2019), but brief details are included below for completeness. Samples were provided by the Fisheries and Oceans, Canada Aquaculture Management Division, Environmental Watch Program, High Seas Program, Strait of Georgia Salmon Program, PARR Program and Salmon Enhancement Program as well as by the Hakai Institute. Hatchery samples are identified by fin clipping, but as not all hatchery fish are marked, wild fish could also encompass unmarked hatchery fish. In this study, we sequenced eighteen Atlantic salmon and twelve Chinook salmon from the Aquaculture Audit for viral discovery and also sequenced one sample from the High Seas Program (B2175) (Supplementary Table S6).

### 2.2 Nucleic acid extractions

DNA was extracted for detection of DNA viruses, bacteria, and parasites from the same tissues from which we extract RNA to target RNA viruses. Nucleic acid extractions on the aquaculture audit samples (eight tissues—gill, atrium, ventricle, liver, pyloric caeca, spleen, head kidney, and posterior kidney) were as previously described (Laurin et al. 2019). For the wild samples, homogenisation using Tri-reagent^TM^ was performed in a Mixer Mill (Qiagen, Maryland) on each tissue independently (five tissues—gill, liver, heart, head kidney, and brain). Tri-reagent^TM^ homogenates were organically separated using bromochloropropane, with the RNA-containing aqueous layer removed for RNA extraction and the lower DNA-containing organic layer separated from the organics using a TNES-Urea Buffer (Asahida et al. 1996).

For the DNA extractions, a pool of 250 μl (five tissues contributing 50 μl each) from each of the tissue TNES aqueous layers were processed for DNA using the BioSprint96 DNA Blood kit (Qiagen, Maryland) and the BioSprint96 instrument (Qiagen, Maryland) based on the manufacturer’s instructions. DNA was quantified using spectrophotometer readings performed on the Infinite M200Pro spectrophotometer (Tecan Group Ltd, Switzerland) and normalised to 62.5 ng/μl using the Freedom Evo (Tecan Group Ltd, Switzerland) liquid-handling unit, based on manufacturer’s instructions.

Similarly, a pool of 100 μl (five tissues contributing 20 μl each) of the aqueous layer was processed for RNA using the Magmax™-96 for Microarrays RNA kit (Ambion Inc., Austin, TX) with a Biomek NXPTM (Beckman–Coulter, Mississauga, ON, Canada) automated liquid-handling instrument, both based on manufacturer’s instructions. The quantity of RNA was analysed using spectrophotometer readings and normalised to 62.5 ng/μl with a Biomek NXP (Beckman–Coulter) automated liquid-handling instrument, based on manufacturer’s instructions. Mixed tissue RNA (1 μg) was reverse transcribed into cDNA using the superscript VILO master mix kit (Invitrogen, Carlsbad, CA) following the manufacturer’s instructions.

### 2.3 VDD biomarkers

The VDD panel of host transcriptional biomarkers is capable of differentiating fish in an active RNA viral disease state from those carrying bacterial or fungal disease (Miller et al. 2017). Biomarkers were derived through meta-analysis of independent published and in-house microarray data and selected for their consistent differential expression upon infection with a range of RNA viral species (Miller et al. 2017). In a previous study (Di Cicco et al. 2018), we derived species-specific VDD thresholds for separating samples with viral disease from samples with no known viral disease using the maximum value of Youden’s J statistic for a receiver operating characteristic analysis of the principal component 1 sample scores. For sequencing, we selected dead and dying farmed Atlantic and farmed Chinook salmon, which exhibited a strong VDD signal, but were deemed negative by RT-PCR for any RNA viruses known to infect salmon in the North Pacific (Piscine orthoreovirus-1, Infectious pancreatic necrosis virus, Infectious haematopoietic necrosis virus, Infectious salmon anaemia virus, and Pacific salmon paramyxovirus).

A statistical model based on the hypergeometric distribution (Spiegel and Stephens 2017) was developed to assess the probability P that a particular novel virus would have been detected via sequencing. The hypergeometric distribution determines the chance that in a pond of *m* infected and *n* non-infected salmon (total *m* + *n*), *x* infected salmon are found when *k* salmon are pulled randomly from the pond. The probability P is equivalent to the chance that at least one infected salmon is found, which can be derived as 1 minus the probability that no infected salmon is found in the *k* randomly pulled salmon. Figure 4C shows probability curves for different virus prevalence as a function of the number *k* of pulled salmon. All curves are based on a total of *m* + *n* = 665 samples as an example, which is the number of Atlantic salmon in the farm audit study. For the curve with a virus prevalence of 10 per cent, parameters *m* = 67 and *n* = 598 are used for the calculation with the hypergeometric distribution.

### 2.4 Metatranscriptomic sequencing

Samples, which were VDD positive but were not positive for any known viruses based on RT-PCR screening (Bass et al. 2019), were prepared for metatranscriptomic sequencing of RNA as previously described (Mordecai et al. 2019). RNA-seq libraries were prepared using the ScriptSeq Complete Epidemiology NGS library kit (Illumina, San Diego, CA), barcoded, and four samples were combined into RNA-seq runs on the Illumina MiSeq platform (Illumina).

Sequences were processed as previously described (Mordecai et al. 2019). In brief, host reads were removed by mapping to the Atlantic salmon genome and unmapped reads were assembled *de novo* using SPAdes (v3.9.1) genome assembler (Bankevich et al. 2012); putative viral sequences were identified by a homology search of translated contigs using DIAMOND (v0.9.16.117) to the nr database using the sensitive mode (Buchfink, Xie, and Huson 2015) as well as HHMER and HHpred searches using the MPI Bioinformatics Toolkit (Supplementary Table S1) (Zimmermann et al. 2018). Assembled sequences are available on GenBank (GenBank accession numbers MN995807–MN995818). In some cases, *de novo* assemblies were extended or scaffolded within Geneious. Assemblies were verified by re-aligning reads to the final scaffold to ensure there was continuous coverage.

### 2.5 Phylogenetic analysis

To infer the phylogenetic relationship of the viruses sequenced from the salmon, the putative viral sequences were compared to sequences obtained from GenBank, which showed similarity to our emerging salmon virus via a translated blast search, as well as using selected sequences from the same viral family to provide context. Sequences with high similarity (greater than 99%) to plant viruses, or host derived sequences, which show similarity to viral sequences (endogenous viral elements) was not included. The evolutionary histories of the viruses were based on the predicted RdRp amino acid sequences, complete coding sequences for ASCV and CTV-2, and partial sequences for the incomplete viral genomes (Supplementary Table S6). Amino acid alignments were generated by MAFFT using the E-INS-i algorithm (Katoh and Standley 2013), retaining all gaps to avoid losing any information. Phylogenetic trees were generated with RaxML to construct ML trees using the PROTGAMMALG model, 100 distinct starting trees and 1,000 bootstraps (Stamatakis 2014). Trees were mid-point rooted for clarity only and annotated within R using ggtree (Yu et al. 2017)*.*  Supplementary Fig. S2 was also generated with RaxML using the GTRCATI model, 100 distinct starting trees and 1,000 bootstraps. The tree was rooted by the outgroup (Avian hepatitis E virus) and viewed in R using ggtree (Yu et al. 2017).

### 2.6 RT-PCR

Assembled viral sequences from the appropriate sample were imported into Primer Express^TM^ v3.0.1 software (Thermo Fisher Scientific, Waltham, MA) where quantitative polymerase chain reaction (qPCR) TaqMan assays were designed using default parameters. High-throughput RT-PCR using these assays was carried out as previously described (Miller et al. 2016; Mordecai et al. 2019), specifically, a 5 μl template mixture was prepared for each sample containing 1× TaqMan Universal Master Mix (No UNG), 1× GE Sample Loading Reagent (Fluidigm PN 85000746) and each of diluted STA’d sample mixtures. Five microlitres of Assay mix was prepared with 1× each of the appropriate TaqMan qPCR assays (agent probe in FAM-MGB and artificial positive construct probe in NED-MGB, 10 μM of primers and 3 μM of probes) and 1× Assay Loading Reagent (Fluidigm PN 85000736). Primer and probe sequences are included in Supplementary Table S5 and the Fluidigm Biomark platform standard TaqMan program for qPCR was used, which includes a hot start followed by 40 cycles at 95 °C for 15 s and 60 °C for 1 min (Fluidigm Corporation, CA, USA). For each assay, a theoretical limit of detection was applied, which results in removing positive detections with a very low load. The limit of detection is set to the concentration of the analyte in the sample matrix that would be detected with high statistical certainty (95% of the time), as previously described (Miller et al. 2016).

### 2.7 Histology and ISH

Fish positive for CTV-2 (*n* = 19), PsNV (*n* = 7), CAV (*n* = 3), and pRNAV (*n* = 2) were selected for histological analysis and ISH to confirm the presence and localisation in the tissues and lesions. Histology samples that tested positive for at least one of the four agents from qPCR detection were used. We used methodologies similar to previously described (Mordecai et al. 2019). Briefly, the fish were euthanized with an overdose of TMS, immediately dissected and the internal organs (gills, skeletal muscle, spleen, liver, heart, anterior and posterior kidney, pyloric caeca, and brain) were fixed in 3.7 per cent neutral buffered formalin. Successively, the tissues were dehydrated through an ascending gradient of ethanol solutions, embedded in paraffin wax, and consecutive serial sections were cut at 3.5-μm thickness. One section per sample was stained with standard haematoxylin and eosin staining for histological examination. The same samples were used to perform ISH staining, which used probes designed to hybridise the RNA of specific viruses. The ISH was implemented using RNAscope^®^ 2.5 HD Duplex Assay (for CAV) and RNAscope^®^ 2.5 HD RED (for CTV-2, PsNV, and pRNAV) (Advanced Cell Diagnostics, Newark, CA, USA) according to the instructions from the manufacturer. In preparation for ISH, consecutive serial dewaxed sections to the ones used for the histopathological analysis were boiled for 30 min in RNAscope target retrieval reagents (Advanced Cell Diagnostics) and then incubated for 30 min in RNAscope Protease Plus reagent prior to hybridisation. The sections were then hybridised with RNAscope Duplex (CAV) or RNAscope^®^ 2.5 HD (CTV-2, PsNV, and pRNAV) probes against a portion of target agent genome segment (Advanced Cell Diagnostics, catalog #513571 [CAV], #513581 [CTV-2], #576651 [PsNV], and #576641 [pRNAV]), to detect the target agent in the tissues. Probes against the bacterial gene DapB were used as a negative control to confirm the absence of background and/or of non-specific cross-reactivity of the assay (Advanced Cell Diagnostics, catalog #310043). Two samples negative through RT-PCR to all the viruses were utilised as negative controls to confirm the absence of cross-reactivity. A probe against the housekeeping gene PPIB (Advanced Cell Diagnostic, catalog #494421 and #540651 for Atlantic salmon and Pacific salmon samples, respectively) was used to assess the quality of RNA present in the tissue sections. The finished histopathological sections went through the first round of visual exam by a veterinary pathologist (co-author E.D.D.), and then were read by a second fish pathologist (co-author H.W.F.). All images captured from the slides were photographed by a camera system (Nikon Digital Sight DS-U3, Nikon, ON, Canada) attached to the Nikon Eclipse Ni microscope (Nikon) and generated by Nikon NIS-Elements D4.30.01 64 Mb software.

## 3. Results

Metatranscriptomic sequencing was carried out on dead and dying farmed Chinook and Atlantic salmon collected as part of the Canadian Department of Fisheries and Oceans aquaculture audit, which samples marine finish aquaculture facilities to meet regulatory requirements. Samples, which exhibited a strong host immune response to viruses (Miller et al. 2017) but were deemed negative by RT-PCR for any RNA viruses known to infect salmon in the North Pacific, were selected for viral discovery. Illumina sequencing followed by *de novo* assembly and a translated similarity search (to the nr database) revealed a collection of novel viruses or novel variants of existing viral species (Supplementary Table S1) including the first detection of Atlantic salmon calicivirus (ASCV) in North America, a new variant of Cutthroat trout virus (CTV-2), and three previously unidentified RNA viruses. For these three viruses, we only obtained partial genome sequences and infection in salmon was unconfirmed so we tentatively named these putative Narna-like virus (pNarnaV), putative toti-like virus (pTotiV), and putative RNA virus (pRNAV). Additionally we confirmed infectivity in salmon of two other recently discovered salmon viruses (Mordecai et al. 2019), Chinook aquareovirus (CAV) and Pacific salmon nidovirus (PsNV).

### 3.1 RT-PCR surveillance of ASCV and CTV-2

**Figure 1.**
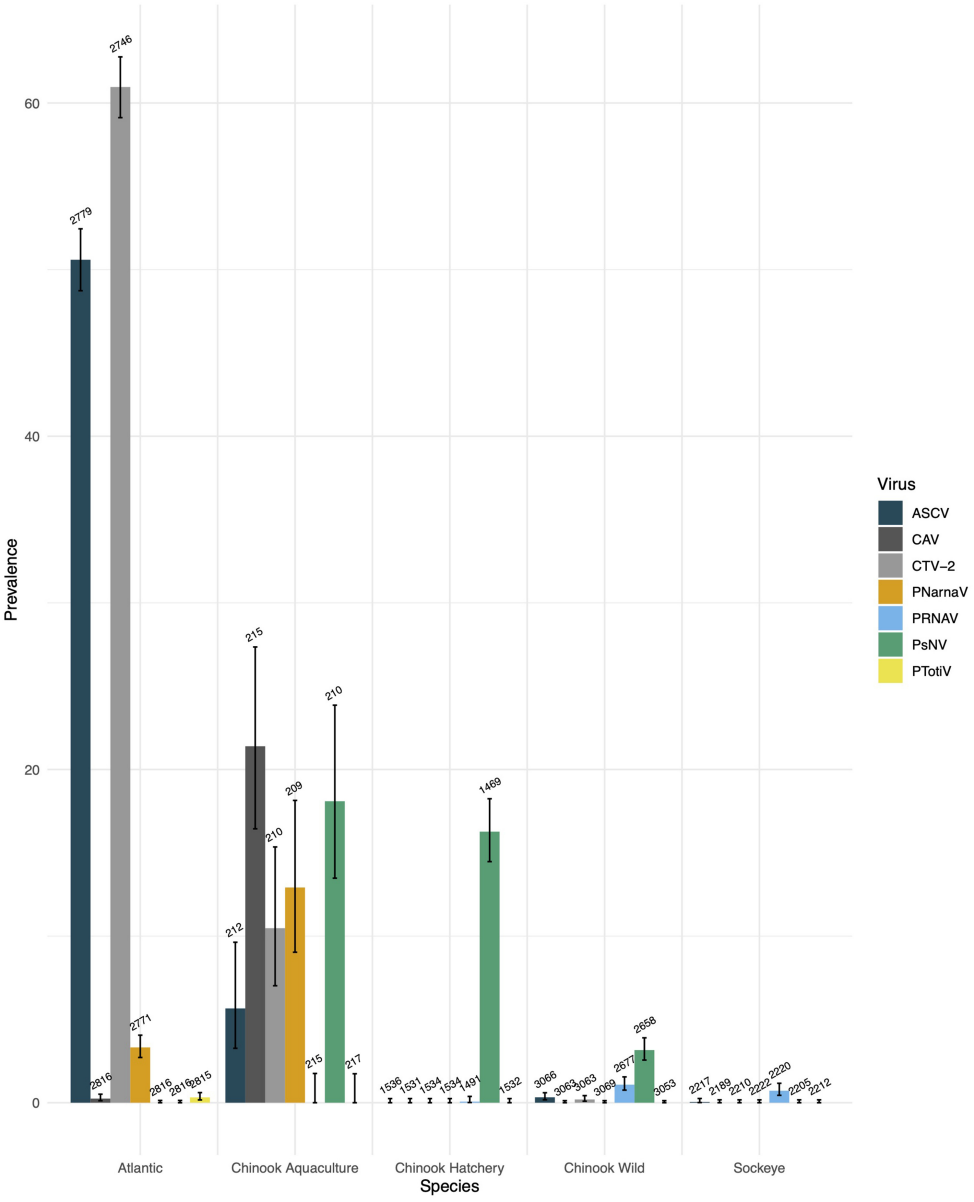
Prevalence (%) of the emerging viruses detected by high-throughput RT-PCR. Numbers show the total number of samples tested, and the error bars show the confidence intervals on the calculated prevalence using the Wilson method. ASCV, CAV, and CTV-2 are all confirmed to infect salmon, whilst the hosts of the pNarnaV, pRNAV, and pTotiV are unconfirmed. Detections of PsNV and CAV in Chinook and sockeye are adapted from Mordecai et al. (2019).

Despite the high prevalence of ASCV in farmed fish in Norway (Wiik-Nielsen et al. 2016), it was only recently reported in Atlantic Canada (Teffer et al. 2020), and prior to the present study, had not been reported from the Pacific, which is surprising considering we found ASCV in over half of the Atlantic salmon tested (Fig. 1 and Supplementary Table S2). Infectivity of ASCV in Atlantic salmon has been shown in Norway (Mikalsen et al. 2014), but its presence in Atlantic salmon in BC is unexplored. Surveillance by high-throughput RT-PCR found that ASCV was common in dead and dying farmed Atlantic salmon (1,406 of 2,779 fish). Interestingly, ASCV was detected in 12 of 212 farmed Chinook and in 10 of 3,066 wild Chinook (Fig. 1).

CTV-2 was common in the salmon aquaculture samples and was detected in 60.1 per cent of farmed Atlantic salmon and 10.5 per cent of farmed Chinook salmon (Fig. 1). However, samples with a high viral load were found in Atlantic salmon only, and these were mostly fish with a positive viral disease biomarker signal.

### 3.2 Phylogenetic relationship of newly discovered viruses in British Columbia

**Figure 2.**
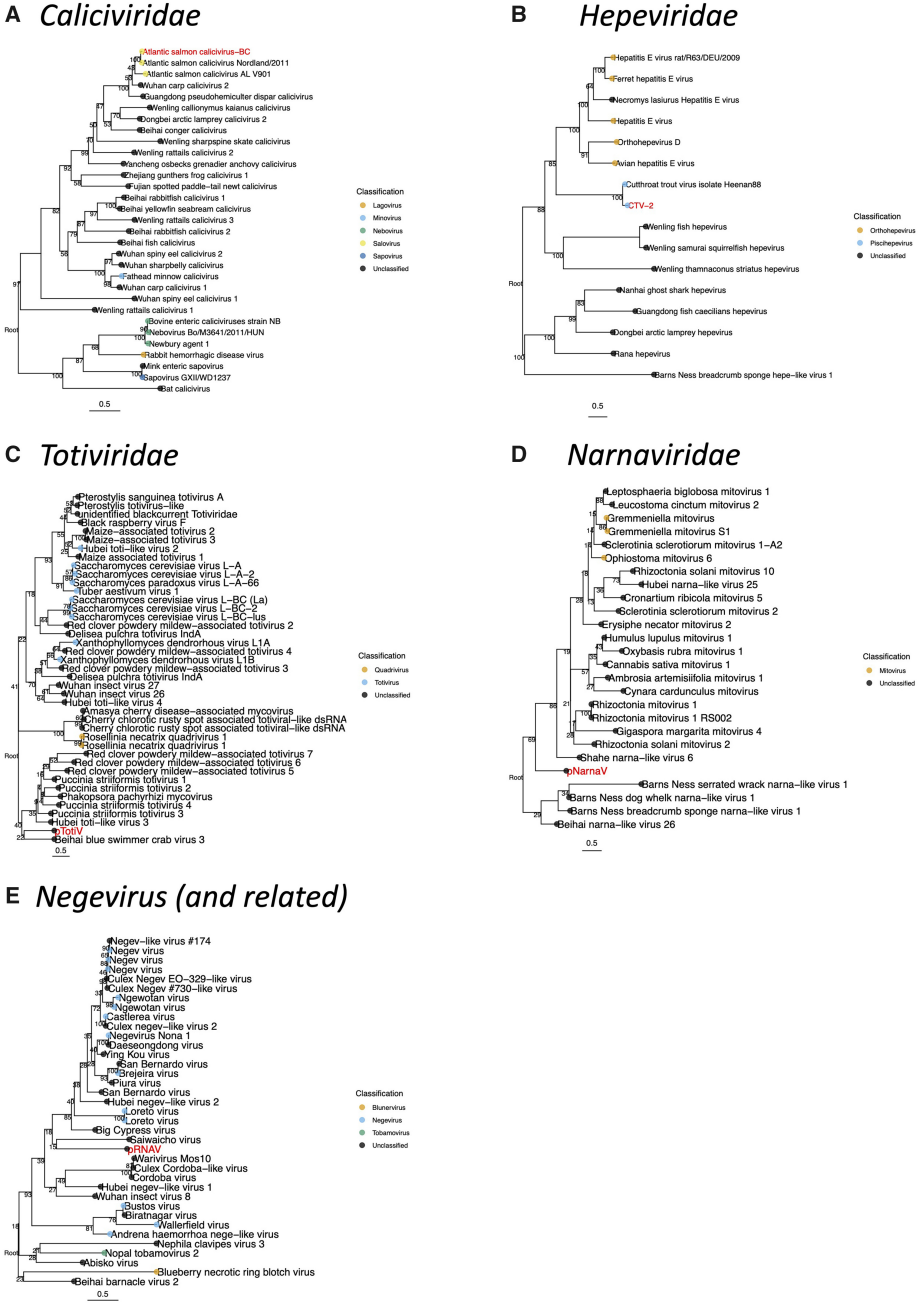
Phylogenetic relationship of (A**)** ASCV, (B**)** CTV-2, (C**)** putative totivirus (pTotiV), (D**)** pNarnaV, and (E**)** pRNAV. Sequences from this study are shown in red. Tip points are coloured by ICTV classification where available. Phylogenies are based on the predicted amino acid RdRp-encoding sequences (CTV-2 phylogeny based on full-length ORF1 polyprotein, see Supplementary Table S7). Branch labels show the bootstrap value, the tree is mid-point rooted for clarity only, and the scale bar shows the mean number of substitutions per site. ASCV and CTV-2 are confirmed to infect salmon, whilst the hosts of the pNarnaV, pRNAV, and pTotiV are unconfirmed.

In this study, the coding-complete genome sequence of ASCV was assembled from a sample taken from a farmed Atlantic salmon (G651). Interestingly, the variant sequenced in farmed Atlantic salmon from BC is more closely related to the Norwegian field variant than the cell-culture isolate sequenced in the same study (Mikalsen et al. 2014) (Fig. 2A). These viruses share 83.8 per cent nucleotide identity and 95.7 per cent amino acid identity (in open reading frame [ORF]1) and form a distinct clade from the cell-culture isolate based on their amino acid sequence. As expected for two closely related viral genomes, the variant we sequenced in BC shares a genome structure similar to those in Norway, with one large ORF1 (7,086 nt), and a second, smaller ORF2 (378 nt), which overlaps with ORF1 in the 3′ end of the genome (Supplementary Fig. S1). ORF2 of the BC ASCV sequence is highly conserved and shares 98.4 per cent amino acid identity with the Norwegian field sequence of ASCV.

We identified a virus genotype closely related to CTV we named CTV-2. Figure 2B shows the phylogenetic placement of CTV-2 within the wider *Hepeviridae* family and in relation to the hepeviruses in fish and frogs recently identified from metatranscriptomic data (Shi et al. 2018). Although many sequence variants of CTV have been partially sequenced (Batts et al. 2011), CTV-2 is phylogenetically distinct from all published sequences (Supplementary Fig. S2). CTV-2 shared 73.7 per cent nucleotide identity (82% amino acid) to the published full-length CTV genome, whereas previous studies found that other variants of CTV had a nucleotide diversity of less than 8.4 per cent (Batts et al. 2011). Notably, CTV-2 is missing a homolog of ORF3 (Supplementary Fig. S1), which is possessed by the first full-length CTV genome (Batts et al. 2011). Predicted ORFs are fragmented in the region where ORF3 is expected.

Previous metatranscriptomic sequencing of aquaculture Chinook revealed a novel reovirus, CAV (Mordecai et al. 2019); phylogenetic analysis of the RNA-dependent RNA polymerase (RdRp) predicted that CAV is part of the genus Aquareovirus, encoding eleven genome segments, each named in accordance with their homology to the grass carp reovirus genome (Supplementary Table S3). All segments are coding complete but lack the terminal conserved untranslated region typical of reoviruses (Mohd Jaafar et al. 2008). Nine of the eleven genome segments were identified by their homology to other aquareoviruses and the presence of conserved reovirus domains (Supplementary Table S4), but there were no hits to the outer fibre or FAST proteins found in other aquareoviruses and orthoreoviruses.

### 3.3 Localisation

**Figure 3.**
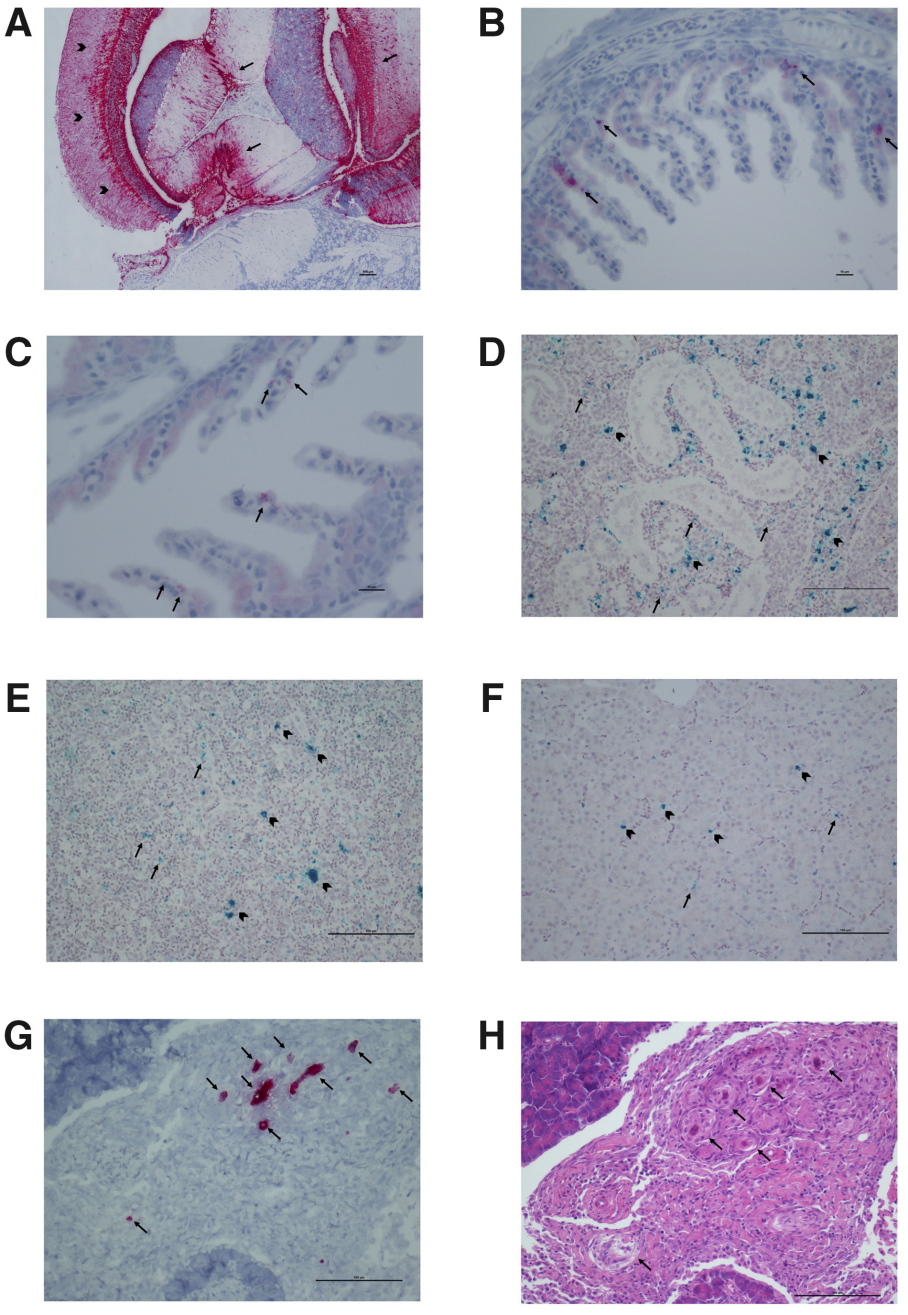
(A) CTV-2 in Atlantic salmon: intense staining (red) localising viral particles in the optic lobes, particularly the stratum griseum, periventricular, and fibrosum profundum (arrowheads) and most of the mesencephalon (arrows). Scale bar 100 μm. (B and C) PsNV in Chinook salmon: viral particles (red, arrows) localised in the epithelial cells of the gills. Scale bar 10 μm. (D) CAV in Chinook salmon: positive marking observed on macrophages (arrowheads) and other cell types in the erythropoietic tissue (arrows). Scale bar 100 μm. (E) CAV in Chinook salmon: widespread marking (green) in the spleen (arrows), particularly in the macrophages (arrowheads). Scale bar 100 μm. (F) CAV in Chinook salmon: positive marking (green) observed in red blood cells (arrows) but also inside some hepatocytes (arrowheads). Scale bar 100 μm. (G) pRNAV in Chinook salmon: serosal surface of intestine showing granulomatous inflammatory response engulfing embryonated metazoan eggs (probably trematodes), marked (red, arrows) for viral particles. Scale bar 100 μm. (H) pRNAV in Chinook salmon: same section as (G), showing granulomatous inflammation (arrows) encompassing several embryonated metazoan eggs (probably trematodes) infected with virus. Scale bar 100 μm.

ISH revealed that CTV-2 infection was systemic, although preferential sites appeared to be the optic lobes of the brain (particularly the stratum griseum periventricular and the stratum fibrosum profundum) and the whole mesencephalon area (Fig. 3A). The same areas were associated with mild neuronal necrosis and degeneration as well as mild ventriculitis and proliferation of ependymal cells. The virus was also observed in the cardiomyocytes (some of which were necrotic), in the splenic ellipsoids and in the renal haemopoietic tissue.

PsNV, first described in a metatranscriptomic survey of Pacific salmon, was shown to be predominantly observed at high prevalence over multiple years in Chinook salmon leaving freshwater hatcheries and was localised to the gills by RT-PCR, reminiscent of the respiratory disease caused by the related mammalian coronaviruses (Mordecai et al. 2019). Here, ISH was used to localise the viral RNA to the epithelial cells of the secondary lamellae of the gills, some of which showed swelling and hydropic degeneration (Fig. 3B and C).

In this study, ISH shows that CAV has a systemic distribution, although it is primarily observed in the splenic, peri-sinusoidal macrophages, and in the renal portal macrophages (Fig. 3D–F). Additionally, scattered hepatocytes show viral particles in the cytoplasm, although lesions were not visible in these cells.

### 3.4 RNA viruses with unconfirmed infectivity in salmon

Finally, smaller fragmented assemblies showed homology to other groups of RNA viruses sequenced from three different samples. Although there was not full genome coverage, we were able to design RT-PCR assays (Supplementary Table S5) for each putative virus to see how widely distributed they were in farmed and wild salmon (Fig. 1). For all three samples, the assay was designed on a *de novo* assembled sequence that showed similarity to the RdRp, enabling phylogenetic analysis (Fig. 2C–E). Although these RdRp sequence fragments were under 500 nt, they are sufficient to reveal phylogenetic relationships of viruses in fish (Geoghegan et al. 2018). Each assembly was translated and aligned with protein sequences obtained from GenBank using search results from BLAST to deduce the phylogenetic relationships of these putative viruses.

On the whole, these viral transcripts were more closely related to invertebrate-associated groups of viruses, implying they were more likely to be infecting invertebrates associated with the fish, rather than the salmon themselves (Geoghegan et al. 2018). For example, several small assembled sequences, referred to in this paper as pRNAV (accession numbers MN995809–MN995814) were detected in a wild Chinook smolt (Sample B2175). These sequences were identified as viral as they showed similarity to a group of arthropod-associated viruses in the proposed *Negevirus* taxa (Fig. 3E) and the arthropod *Iflavirus* genus. Additionally, screening by high-throughput RT-PCR revealed that this virus was detected in only 0.69 per cent of Chinook and sockeye salmon (forty-six of 6,603 samples) (Fig. 1). Here we show, using ISH, that this putative virus is not an environmental contaminant but appears to be associated with an invertebrate parasite of the salmonid host, raising the possibility that there is an alternative invertebrate host for this virus. The virus was primarily observed in structures (tentatively identified as embryonated metazoan eggs, possibly trematode eggs) encysted in the lamina propria of the intestine and localised on the serosal surface (Fig. 3G and H). The putative eggs were surrounded by a granulomatous inflammatory reaction. Viral RNA was also detected in the exocrine pancreas and peri-capsular adipose tissue of the spleen.

Additionally, we identified sequences with similarity to viruses belonging to the *Narnaviridae*, a family of viruses that lack a capsid, which we named pNarnaV, due to its phylogenetic placement (Fig. 2D). Narnaviruses are non-infectious RNA transcripts that are assumed to be vertically transmitted and which reproduce inside the mitochondria (genus *Mitovirus*) or in the cytosol (genus *Narnavirus*) of the host (Hillman and Cai 2013; Dolja and Koonin 2018). pNarnaV was detected solely in aquaculture samples, in both Atlantic and Chinook salmon (3% and 13% prevalence, respectively). Additionally, a small sequence assembly (309 nt) with similarity to a variety of fungi-infecting totiviruses was detected in farmed Atlantic salmon and named pTotiV (Figs 1 and 2C). However, the prevalence of pTotiV was extremely low, with just nine out of 2,815 (0.3%) samples of farmed Atlantic salmon samples positive for the virus, and no positive detections in 4,802 samples of Chinook salmon.

### 3.5 VDD biomarkers and virus discovery

**Figure 4.**
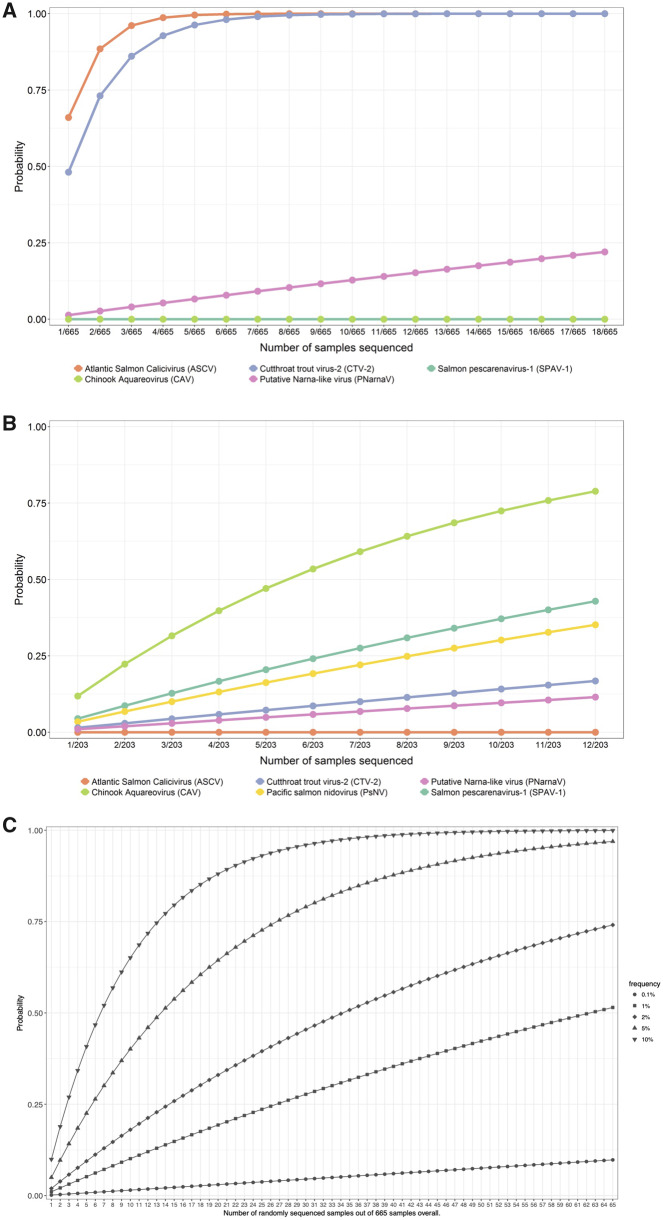
(A) Probability of finding the specified virus when sampling *k* samples randomly from the set of 665 Atlantic salmon in the farm audit data and (B) when sampling *k* samples randomly from the set of 203 Chinook salmon in the farm audit data. A copy number threshold of 1,000 was used to define detection. The *x*-axis goes up to values of *k* = 12 for Chinook salmon and *k* = 18 for Atlantic salmon, which were the number of samples sequenced based on suggestions derived from VDD patterns in the audit data sets. (C) Probability of detecting a novel virus in 665 samples by randomly sequencing the specified subset of samples. Curves represent different scenarios where the specified percentage of 665 samples overall has a theoretical detectable viral load for NGS sequencing.

To demonstrate the effectiveness of sample selection via the VDD biomarker, we calculated the probability of detecting novel or emerging viruses if we had chosen a random subset of the aquaculture audit samples available for sequencing. We include newly described viruses, which were previously detected in farmed Chinook including Salmon pescarenavirus-1 (SPAV-1), CAV, and PsNV (Mordecai et al. 2019). Figure 4A and B shows curves for the probability that randomly sequencing *k* samples from the aquaculture audit would lead to virus detection in at least one salmon. In these plots, observed detections by RT-PCR in the aquaculture data sets were used to define prevalence (Supplementary Data). Only viruses that had positive detections in the aquaculture audit for each species were included. A theoretical copy number threshold of 1,000 nucleic acid copies per µg of tissue was used to estimate the chance of identifying a novel virus in a metatranscriptomic sequencing run. With this threshold, five of the viruses were detected in the Chinook salmon audit data (9/203 with SPAV-1, 7/203 with PsNV, 3/203 with CTV-2, 2/203 with pNarnaV, and 24/203 with CAV) and three of the viruses were detected in the Atlantic salmon audit data (439/665 with ASCV, 320/665 with CTV-2, and 9/665 with pNarnaV). Both ASCV in Chinook salmon and SPAV-1 and CAV in Atlantic salmon had positive detections but were below the copy number threshold of 1,000; thus, the corresponding curves show probabilities of zero. Similarly, the probability of detecting a novel virus depends on how many samples were sequenced and the prevalence of the virus in the population (Fig. 4C). The frequency is based on NGS-detectable infections, i.e. if 1,000 fish were sequenced and 100 were positive for a specific virus, the prevalence would be defined as 10 per cent. pTotiV was first discovered in an alternative aquaculture data set (Di Cicco et al. 2018), which was not part of the Canadian Department of Fisheries and Oceans aquaculture audit, so is excluded from this analysis. The probabilities described in Fig. 4 demonstrate that for more widely occurring viruses, a targeted sequencing approach is not necessary; whereas, the rarer viruses would likely remain undiscovered without our VDD-based sample selection approach.

## 4. Discussion

In this study, we report the first detection of ASCV in North America, as well as a new variant of CTV, and find that these viruses are highly prevalent in farmed Atlantic salmon in BC and are also detected in Chinook salmon. We show that the combination of the VDD biomarker panel combined with metatranscriptomic sequencing, RT-PCR surveillance, and ISH is appropriate tools for viral discovery.

The number of viral transcripts in fish metatranscriptomes is hugely outnumbered by host transcripts and other contaminants (Supplementary Table S6) (Geoghegan et al. 2018; Zhang et al. 2019). Therefore to achieve full genome coverage, high depth of sequencing is required. In viral discovery studies, there is a trade-off between sequencing a larger number of samples at a lower depth of coverage versus less samples at a higher depth. If coverage is too low, viral genome assembly can be fragmented and only regions of the genome with similarity to known viruses are detected whilst others could be missed entirely. As a compromise, our strategy was to sequence a larger number of VDD-positive samples at a relatively low depth. Although this did not yield ‘coding-complete’ sequences for all the putative novel viruses, we were able to sequence enough of the viral genome to design RT-PCR primers to inform a high-throughput epidemiological survey in which we screened over 9,000 salmon sampled over one decade along the coast of Southern BC (Fig. 1), conduct phylogenetic analyses (Fig. 2), as well as design probes for ISH to determine infectivity and tissue tropism (Fig. 3).

ASCV was the first calicivirus in fish to be fully characterised and sequenced (Mikalsen et al. 2014). ASCV was detected in a high proportion of farmed Atlantic salmon in Norway and was observed in association with several diseases including heart and skeletal muscle inflammation. However, ASCV was also commonly found in healthy fish and a more recent study found no correlation between ASCV and heart and skeletal muscle inflammation (Mikalsen et al. 2014; Wiik-Nielsen et al. 2016). However, there is evidence that co-infection of caliciviruses with other viruses could be linked to clinical manifestations of disease in baitfish (Mor et al. 2017). In this study, in farmed Atlantic salmon from BC, we discovered viral genomes extremely closely related to the ASCV genome first discovered and sequenced in Norway (Mikalsen et al. 2014).

The initial discovery of ASCV in Norway (Mikalsen et al. 2014) revealed two variants that shared 70.9 per cent nucleotide identity across the whole genome, one of which was sequenced directly from field material whilst the other was isolated through cell culture and subsequently sequenced. This suggests that the cell-culture isolate may have undergone selection pressure under culture conditions, which is plausible considering it was cultured in the GF-1 cell line, which originates from grouper, *Epinephelus coioides* (Chi, Hu, and Lo 1999). We found that ASCV in BC and the wildtype sequence in Norway are closely related, whilst the cell line isolate is the most divergent of these three sequences.

Caliciviruses are widespread in fish, and although we know very little of their direct role in lesion development, caliciviruses have been associated with diseased fish in aquaculture (Mor et al. 2017). Phylogenetically, caliciviruses are part of the picorna-like virus group, which is thought to have evolved since eukaryogenesis (Koonin et al. 2008), so it is not surprising that they are found extensively in all groups of vertebrates and cause a wide variety of diseases in mammals and birds (Wolf et al. 2009; Tse et al. 2012; Scheuer et al. 2013; Lefkowitz et al. 2018), including transient clinical diseases as well as haemorrhaging and mortality (Smith et al. 1998; Cooke 2002; Hurley et al. 2004). Interestingly, some caliciviruses infect marine mammals (Smith and Boyt 1990), and at least one fish species has been reported as a reservoir for vesicular exanthema of swine virus, which can infect and cause disease in mammals (Smith et al. 1980, 1981). More recently caliciviruses have been discovered in several fish and amphibia (Mikalsen et al. 2014; Mor et al. 2017; Shi et al. 2018). Phylogenetically, fish caliciviruses are basal to mammalian caliciviruses (Shi et al. 2018), and ASCV groups within a clade distinct from the mammalian caliciviruses (Fig. 2A), and are more closely related to metatranscriptomic discovered fish caliciviruses (Shi et al. 2018) and fathead minnow calicivirus (Mor et al. 2017). Fish caliciviruses (Mor et al. 2017; Shi et al. 2018) are phylogenetically distinct from those in mammals, suggesting that spillover from fish to mammals, which has been previously reported (Smith et al. 1980, 1981), is not a common occurrence. Due to the sequence similarity of ASCV in BC to the Norwegian variant, and its high prevalence in farmed Atlantic salmon in BC, it seems plausible that ASCV originates from Norwegian Atlantic salmon that were introduced to BC for aquaculture. Detection of ASCV in Chinook suggests a broad host range and these findings are corroborated by replication of ASCV in the grouper cell line. Once introduced to a new region, transmission between populations and species is feasible considering caliciviruses remain viable even after exposure to sea water for 14 days (Smith et al. 1998).

In the past decade, there has been a surge in discovery of Hepatitis E viruses in various animals (Doceul et al. 2016), including CTV, the first hepevirus identified in fish (Dalton et al. 2008; Batts et al. 2011; Drexler et al. 2012). We hypothesise that CTV-2 represents a second lineage of CTV adapted to salmonid species. CTV was first isolated in the CHSE-214 (Chinook salmon embryo) cell line, from material collected from cutthroat trout (*Oncorhynchus clarkia*) (Batts et al. 2011), and is the first member of the genus *Piscihepevirus* (Meng 2013; Smith et al. 2014). In recent years, a range of hepeviruses have been discovered in fish metatranscriptomes (Shi et al. 2018). These viruses are phylogenetically diverse and are likely representative of other unclassified hepevirus genera, which infect fish. Here we discovered a viral sequence with similarity to CTV, which we named CTV-2, due to a relatively high sequence similarity and overlapping host range. To date, CTV has not been associated with disease but it is widespread in trout populations in the Western USA and has also been detected in Atlantic salmon (Kibenge et al. 2000; Batts et al. 2011). Interestingly, although CTV is widespread in North America, it has not been detected on any other continent (Batts et al. 2011), which suggests this may represent a transmission event from a Pacific species to Atlantic salmon. However, a closely related virus has been detected in Atlantic Canada (Kibenge et al. 2000), so the origin is not clear. Similar to ASCV, detection of this virus in Chinook highlights the potential for cross-species transmission.

Interestingly, CTV-2 lacks ORF3, which is found in other hepeviruses, and encodes an immunogenic protein, which is thought to be involved in virion morphogenesis and pathogenesis, but its role is not fully understood. ORF3 is not highly conserved amongst hepeviruses and there is a low similarity in ORF3-encoding sequences between viruses from different hosts, suggesting it represents viral adaptation to a particular host (Drexler et al. 2012). Furthermore, hepeviruses recently sequenced in fish are also missing ORF3 (Shi et al. 2018).

We used ISH to demonstrate infection in salmon by two viruses (PsNV and CAV) described in a previous metatranscriptomic study (Mordecai et al. 2019). Our results confirm the previous finding via RT-PCR that PsNV primarily infects gill tissue. This finding supports our previous hypothesis that disease caused by the virus is manifested when fish undergo physical stress due to osmotic imbalance as they move from fresh to marine waters during smoltification (Houde et al. 2019; Mordecai et al. 2019). Additionally, we show that CAV infection is systemic, although it was primarily localised to macrophages. It is unclear if CAV infects these immune cells as a ‘Trojan horse’ and mediates virus spread in the whole organism whilst being protected from the host immune response (Cafruny and Bradley 1996), or whether the virus was simply localised in macrophages following phagocytosis in an effort to eliminate the virus from the blood.

CAV can be grouped with a growing number of aquareoviruses, which are phylogenetically basal to those first discovered in fish (Nibert and Duncan 2013; Mordecai et al. 2019). This group includes Hubei grass carp reovirus (HGCRV, also known as GCRV104) (Fan et al. 2013) and Pangasius aquareovirus (unpublished, sequence available on GenBank) and the Western African Lungfish Reovirus (Shi et al. 2018). Of these, HGCRV is the most studied, as it causes haemorrhagic diseases of grass carp and serious loss to carp aquaculture in China (Wang et al. 2012). Interestingly, the addition of these metatranscriptomic sequences (Fan et al. 2013; Shi et al. 2018) further breaks down the shallow distinction between aquareoviruses and orthoreoviruses, lending weight to the call for these genera to be merged (Nibert and Duncan 2013). CAV is more akin to the aquareovirus group due to its phylogenetic placement based on the RdRp, as well as possessing eleven genome segments, each of which appear to encode an ORF. As segments seven and eleven showed no recognisable sequence similarity using a translated BLAST of the RefSeq non-redundant database (O’Leary et al. 2016), these sequences were attributed to CAV by their size, predicted ORFs, and their primary protein structure. Further analysis of these predicted proteins revealed features reminiscent of closely related reoviruses. For example, HHpred analysis of segment 7 revealed a predicted protein domain similar to the orthoreovirus sigma-1 protein and adenovirus fibre proteins, suggesting it encodes the fibre protein that is present in some, but not all aquareoviruses and Piscine orthoreovirus (Nibert and Duncan 2013). Similarly, the putative segment 11 of CAV, contains a signal peptide and transmembrane domains as detected by Phobius (Käll, Krogh, and Sonnhammer 2007) (Supplementary Fig. S3). These observations are similar to those of HGCRV, Piscine orthoreovirus and GCRV-HZ08/GD10, which also contain transmembrane domains that could represent a membrane interacting non-structural protein (Nibert and Duncan 2013).

In this study, we partially sequenced viral genomes, which resembled invertebrate viruses. pRNAV was localised to an invertebrate parasite as well as in the spleen of the salmon. Recent evidence of arthropod viruses circulating in the blood of a vertebrate host (Bennett et al. 2019) adds to the growing number of instances where arthropod viruses have been detected in vertebrates, suggesting that cross-kingdom transmission could be more common than previously thought. However, in many of these cases, infectivity has not been confirmed, and detection of contaminating sequences from alternative hosts remains a possibility. Similarly, there have been reports of novel flaviviruses infecting both marine vertebrates and invertebrates (Parry and Asgari 2019). It is not clear if the localisation of pRNAV in the spleen reflects the dissemination of the virus from the metazoan eggs in the intestine, or infection of an immature stage of the parasite itself. The detection in fish of viruses that phylogenetically resemble viruses of invertebrates reflects recent work, which found sequence from the same virus in both crabs and sharks (Parry and Asgari 2019). It is apparent that in the marine environment, certain groups of viruses are transmitted horizontally between invertebrates and vertebrates, in which case distinction between groups of viruses that infect vertebrates and invertebrates is starting to break down.

Similarly, we report the detection of sequences with similarity to the *Narnaviridae.* These viruses are known to infect fungi and are widespread in the viromes of plant-pathogenic fungi (Marzano et al. 2016). This group was greatly expanded in a recent metatranscriptomic study of invertebrates (Shi et al. 2016), and many of these viruses appear to originate from host-associated fungi, although it has been suggested that the sheer number and diversity of this group of viruses implies a broader host range (Dolja and Koonin 2018). It is unclear if the pNarnaV infected the salmon, or an associated fungal parasite, but as the sequence was only found in dead salmon, it suggests that the virus may be associated with fungi associated with decomposing fish.

Low detections of pTotiV meant that it was not possible to determine the host range of this virus. There is a developing body of work demonstrating that within one viral family, cross-kingdom host ranges can occur (Li et al. 2014; Coyle et al. 2018). Originally, totiviruses were thought to only infect unicellular fungi, but the known host range of totiviruses has expanded to include arthropods and fish (Haugland et al. 2011; Koyama et al. 2015). Pertinently, a totivirus, piscine myocarditis virus (PMCV), was shown by ISH to infect Atlantic salmon and is associated with cardiomyopathy syndrome (Løvoll et al. 2010; Haugland et al. 2011). Additionally, a totivirus has been implicated as an emerging pathogen in paenid-shrimp aquaculture (Tang et al. 2008; Prasad et al. 2017). The lack of sequence similarity between pTotiV and PMCV implies that pTotiV is evolutionarily distinct from PMCV and may represent a second evolutionary lineage of totiviruses infecting vertebrates. These findings highlight the difficulties in identifying the host of novel viruses discovered in metatranscriptomic surveys of RNA viruses purely based on sequence alone.

## 5. Conclusions

In this study, we used ISH, sequencing, and RT-PCR surveillance to show that newly discovered viruses ASCV and CTV-2 occur in Atlantic salmon in BC and are also detected in Chinook salmon. These viruses have phylogenetic proximity to other viruses that infect fish, suggesting that they may be important to salmon health. However, the detection of RNA via RT-PCR does not demonstrate infection, and in some cases novel virus sequences could represent a virus from an alternative host associated with salmon, such as pRNAV, which was seen in embryonated eggs of what was likely a trematode parasite. Similarly, the hosts of the putative viruses, pTotiV and pNarnaV, are not clear, and they may have no relevance to investigations of salmon health.

Disease is a factor affecting rates of population mortality, and infectious diseases are an integral part of ecosystems. However, the presence of a virus is not synonymous with disease; infections can be acute, subacute, chronic, or inapparent (Naish et al. 2007). However, changing environmental or anthropogenic factors, for instance, the introduction of a vector (Martin et al. 2012), an alternative host or high density rearing environments (Kennedy et al. 2016), can facilitate rapid viral propagation and result in increased evolutionary pressure for the emergence of more virulent strains or enable host-range expansion. Therefore, to understand the mechanisms of viral emergence, we need to characterise virus diversity amongst different host species. Moreover, environmental stressors such as climate change could result in disease emergence (Pagowski et al. 2019; Price et al. 2019), even from agents that might have previously been tolerated. Therefore, continual discovery and surveillance of emerging viruses in ecologically important salmon will be vital for management of both aquaculture and wild resources in the future.

Previously, we identified VDD-positive fish with no known viruses based on RT-PCR screening (Di Cicco et al. 2018) and selected these for sequencing. Three previously unknown viruses were identified from eighteen Atlantic salmon, and four more viruses were identified in twelve Chinook salmon. For an assumed, required copy number threshold of 1,000 to detect viruses by NGS on a MiSeq, the probability of detection of pNarnaV in Atlantic salmon was 22 per cent if fish were randomly selected. ASCV and CTV-2 would have been detected with 100 per cent certainty. For Chinook salmon, the probability of pNarnaV, CTV-2, PsNV, SPAV-1, and CAV was 11.5 per cent, 16.8 per cent, 35.2 per cent, 42.9 per cent, and 78.9 per cent if fish were sampled randomly. In the future, the VDD panel will be an important tool to enable the discovery of viruses in wild salmon, in which we expect to find fewer positive or high-load samples than in farmed fish. Moreover, given the high overlap in genes contributing to the salmon VDD panel and those discovered to be predictive of respiratory viral diseases in humans (Zaas et al. 2009; Andres-Terre et al. 2015), there is a high potential that a similar panel of genes could be applied for viral discovery in a diverse range of vertebrate host species (Miller et al. 2017).

## Supplementary data

Supplementary data are available at *Virus Evolution* online.

## Supplementary Material

veaa069_Supplementary_Data
