# Supplementary material for: Discovery and surveillance of viruses from salmon in British Columbia using viral immune-response biomarkers, metatranscriptomics, and high-throughput RT-PCR
Source: Virus Evol. 2020 Sep 1;7(1):veaa069. doi: 10.1093/ve/veaa069 (PMC7887441; doi:10.1093/ve/veaa069)
Supplement: veaa069_Supplementary_Data [file veaa069_supplementary_data.pdf]

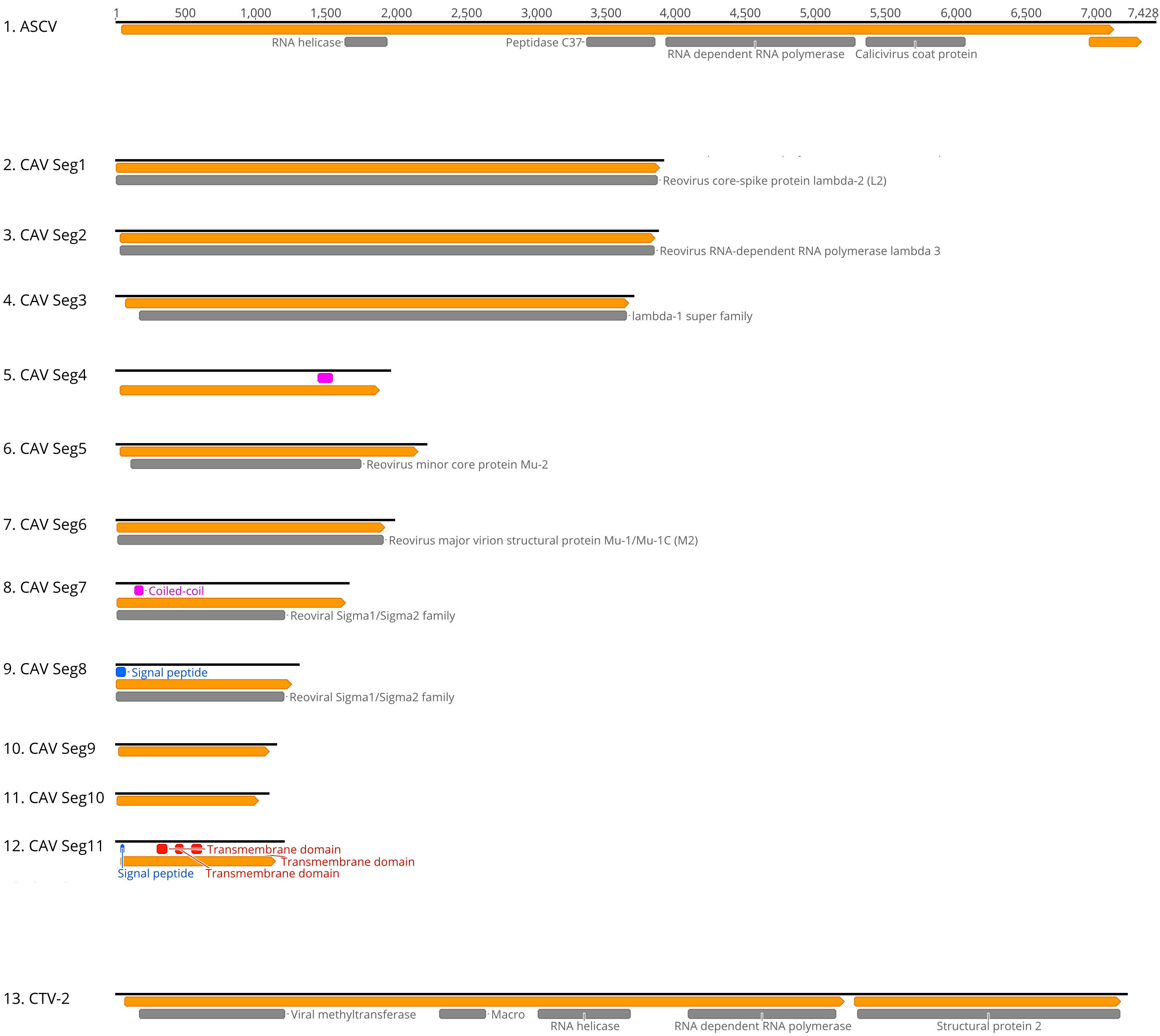

Supplementary figure 1 Overview of the genome structure of the emerging viruses for which we sequenced coding complete genomes; Atlantic Salmon Calicivirus (ASCV), Chinook Aquareovirus (CAV) segments 1-11, and Cutthroat trout virus-2 (CTV-2). Black lines represent the genome, orange boxes show the predicted ORFs with conserved protein domains shown in grey. Blue boxes indicate the detection of a leader protein, pink boxes show predicted coiled-coil domains, and red boxes show predicted transmembrane domains.

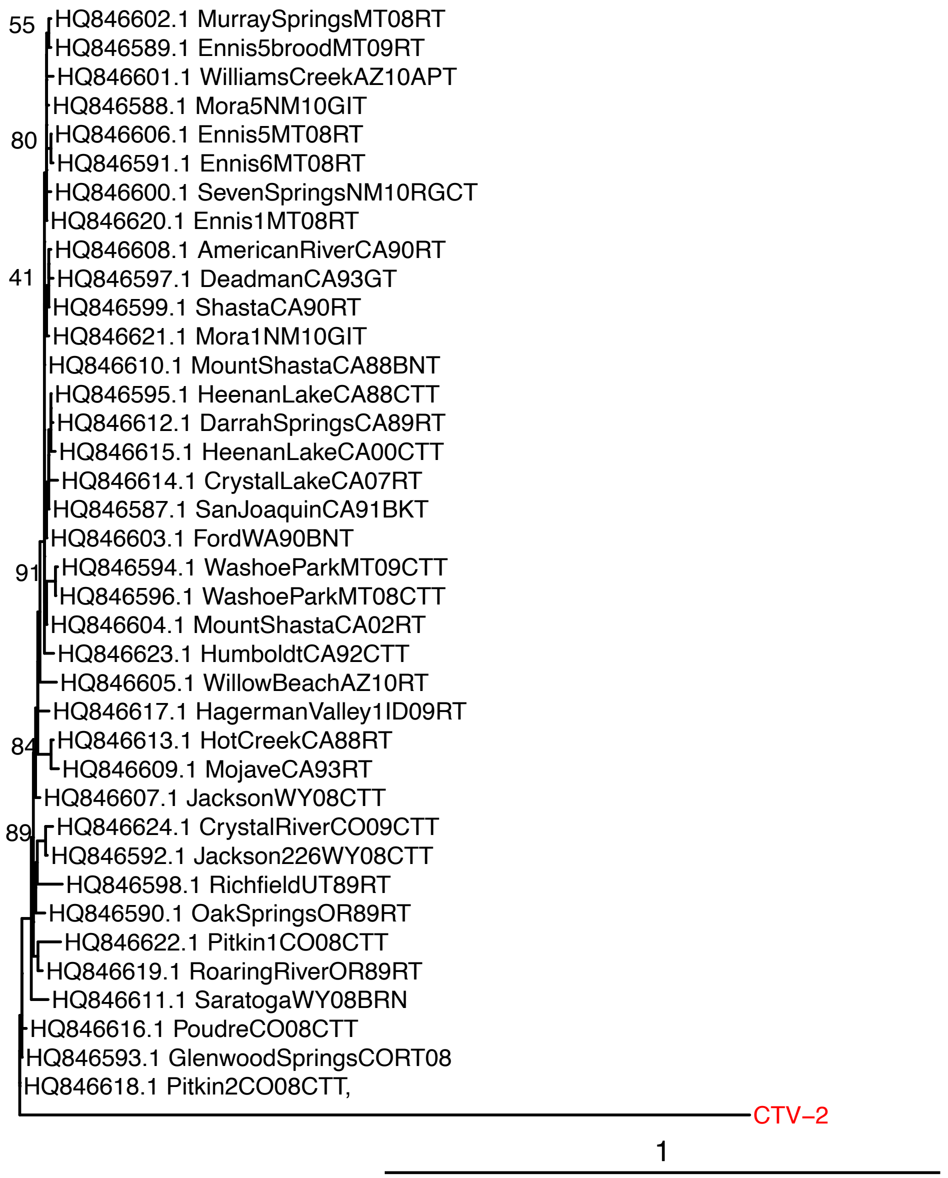

Supplementary figure 2 Phylogenetic relationship of Cutthroat trout virus based on a portion of the helicase nucleotide sequence. Scale bar shows the mean number of nucleotide substitutions per site. Node numbers show the bootstrap values above 40. The sequence from this study is shown in red. The tree was rooted by the outgroup (Avian hepatitis E virus) which was then removed for viewing the tree.

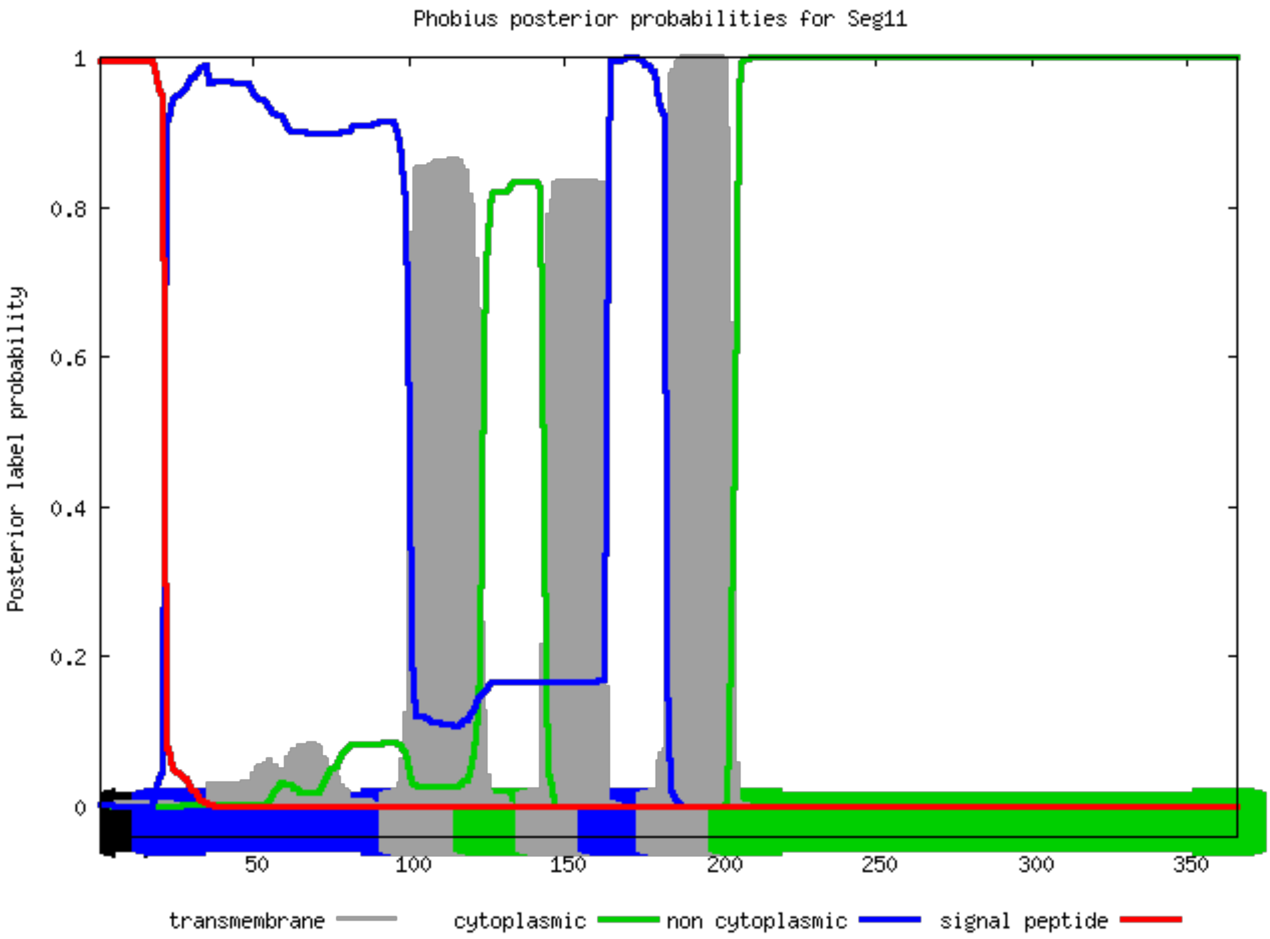

Supplementary figure 3 Phobius output for the predicted protein encoded by Segment 11 of Chinook Aquareovirus (CAV)

Virus species	Genbank accession	Length (nt)	Coding complete	Top hit (DIAMOND)	Percentage identity	Infection in fish shown by ISH
Atlantic salmon calicivirus BC	MN995807	7426	Yes	YP 009026987.1 polyprotein [Atlantic salmon calicivirus]	90.0	Yes
Cutthroat trout virus-2	MN995808	7127	Yes	YP 004464917.1 polyprotein [Cutthroat trout virus]	82.8	Yes
Putative Narna-like virus contig 1	MN995815	335	No	ASM94097.1 putative RNA-dependent RNA polymerase, partial [Barns Ness serrated wrack narna-like virus 1]	35.1	No
Putative Narna-like virus contig 2	MN995816	300	No	ASM94097.1 putative RNA-dependent RNA polymerase, partial [Barns Ness serrated wrack narna-like virus 1]	38.1	No
Putative RNA virus contig 1	MN995809	190	No	YP 009344984.1 RNA-dependent RNA polymerase [Xinzhou nematode virus 5	73.8	No
Putative RNA virus contig 2	MN995810	214	No	YP 009344984.1 RNA-dependent RNA polymerase [Xinzhou nematode virus 5	73.2	No
Putative RNA virus contig 3	MN995811	224	No	AQM55302.1 hypothetical protein 1 [Cordoba virus]<>AQM55303.1 hypothetical protein 1 [Cordoba virus]	53.4	No
Putative RNA virus contig 4	MN995812	225	No	YP 009344994.1 hypothetical protein [Wuhan insect virus 8]<>APG77763.1 hypothetical protein [Wuhan insect virus 8]	36.8	No
Putative RNA virus contig 5	MN995813	289	No	ARA91660.1 polyprotein [Sugar beet cyst nematode virus 1]	39.8	No
Putative RNA virus contig 6	MN995814	391	No	AQM55317.1 hypothetical protein 1 [Ngewotan virus]	37.5	No
Putative toti-like virus contig 1	MN995817	292	No	ADQ54106.1 RNA polymerase [Tuber aestivum virus 1]	52.9	No
Putative toti-like virus contig 2	MN995818	309	No	ATO91009.1 putative RNA-dependent RNA polymerase, partial [Puccinia striiformis totivirus 2]	50.0	No

Supplementary table 1. Summary of newly discovered and emerging viruses.

Virus	Host Population	Number of samples tested	Number of positive samples
ASCV	Chinook Aquaculture	212	12
ASCV	Chinook Hatchery	1536	0
ASCV	Chinook Wild	3066	10
ASCV	Sockeye	2217	1
ASCV	Atlantic	2779	1406
CAV	Chinook Aquaculture	215	46
CAV	Chinook Hatchery	1531	0
CAV	Chinook Wild	3063	0
CAV	Sockeye	2189	0
CAV	Atlantic	2816	7
CTV-2	Chinook Aquaculture	210	22
CTV-2	Chinook Hatchery	1534	0
CTV-2	Chinook Wild	3063	6
CTV-2	Sockeye	2210	0
CTV-2	Atlantic	2746	1674
pNarnaV	Chinook Aquaculture	209	27
pNarnaV	Chinook Hatchery	1534	0
pNarnaV	Chinook Wild	3069	0
pNarnaV	Sockeye	2222	0
pNarnaV	Atlantic	2771	92
pRNAV	Chinook Aquaculture	215	0
pRNAV	Chinook Hatchery	1491	1
pRNAV	Chinook Wild	2677	29
pRNAV	Sockeye	2220	16
pRNAV	Atlantic	2816	0
PsNV	Chinook Aquaculture	210	38
PsNV	Chinook Hatchery	1469	239
PsNV	Chinook Wild	2658	84
PsNV	Sockeye	2205	0
PsNV	Atlantic	2816	0
pTotiV	Chinook Aquaculture	217	0
pTotiV	Chinook Hatchery	1532	0
pTotiV	Chinook Wild	3053	0
pTotiV	Sockeye	2212	0
pTotiV	Atlantic	2815	9

Supplementary table 2 RT-PCR surveillance of viruses. Detections of PsNV and CAV in Chinook and sockeye are adapted from Mordecai et al. (2019).

Segment	GC%	Length (nt)	Length (aa)	Top hit
Seg-1	50.4086	3916	1292	GCRV104 VP1 Core Turret
Seg-2	48.7232	3877	1272	GCRV104 VP2 Core RdRp
Seg-3	50.1757	3699	1198	GCRV104 VP3 Core shell
Seg-4	53.1091	1962	617	GCRV104 VP66 NS factory
Seg-5	51.0791	2224	708	GCRV104 VP5 Core NTPase
Seg-6	48.8956	1992	638	GCRV104 VP4 Outer shell
Seg-7	48.5012	1668	543	No homology – 'putative fiber protein'
Seg-8	50.3817	1310	417	GCRV104 VP6 Core clamp
Seg-9	50.6957	1150	358	GCRV RNAB
Seg-10	51.8282	1094	336	GCRV104 outer clamp
Seg-11	50.6633	1206	366	No homology – 'putative NS other'

Supplementary table 3. Genome segments encoded by Chinook aquareovirus (Mordecai et al. 2019) and homology to Grass carp reovirus (GCRV104)

Virus	Predicted Protein function/ structure	Region start (nt)	Region end (nt)
ASCV	RNA helicase	1641	1939
ASCV	Peptidase C37	3366	3853
ASCV	Calicivirus coat protein	5363	6069
ASCV	RNA dependent RNA polymerase	3933	5285
CAV Seg1	Reovirus core-spike protein lambda-2 (L2)	7	3869
CAV Seg2	Reovirus RNA-dependent RNA polymerase lambda 3	34	3848
CAV Seg3	lambda-1 super family	174	3650
CAV Seg4	Coiled-coil	1448	1548
CAV Seg5	Reovirus minor core protein Mu-2	112	1753
CAV Seg6	Reovirus major virion structural protein Mu-1/Mu-1C (M2)	16	1912
CAV Seg7	Coiled-coil	138	196
CAV Seg7	Reoviral Sigma1/Sigma2 family	11	1209
CAV Seg8	Signal peptide	8	71
CAV Seg8	Reoviral Sigma1/Sigma2 family	8	1202
CAV Seg11	Signal peptide	41	58
CAV Seg11	Transmembrane domain	431	486
CAV Seg11	Transmembrane domain	548	614
CAV Seg11	Transmembrane domain	300	369
CTV-2	Macro	2315	2643
CTV-2	RNA helicase	3021	3679
CTV-2	Viral methyltransferase	174	1212
CTV-2	RNA dependent RNA polymerase	4094	5144
CTV-2	Structural protein 2	5301	7177

Supplementary table 4 Predicted protein functions or structure for the coding complete genomes of ASCV-BC, CAV and CTV-2. The region of homology as predicted by HMMER are shown by the coordinates on the corresponding nucleotide sequence.

Target virus	Forward Primer Sequence (5'-3')	Probe Sequence (5'-3')	Reverse Primer Sequence (5'-3')	Assay size (bp)
ASCV	ACCGACTGCCCGGTTGT	CTTAGGGTTAAAGCAGTCG	CTCCGATTGCCTGTGATAATACC	81
CTV-2	CCACTTGTCGCTACGATGAAAC	ATGCCGGGCCATC	CGCCTCCTTTGCCTTTCTC	75
PTotiV	TCTGCGCGCTGCACCTA	CAAGTGCTACACTGCG	ATGCGGAGGAACTCACACACT	57
pNarnaV	TGTCCCTGAAGATTCATTTCGA	TCCTAGGTGATGATATAAT	CTATGTAAAGCCTCGTCGGTGAT	66
pRNAV	GTACCTAATTTAACTGGAACAGTAGAC	TGCAACAGGCAAGTGATATGCTTGA	CGTTCAGTAACACAAGTATCCAAA	86

Supplementary table 5 Taqman assays designed and used in this study.

Target Virus	Library Name	Sample collection	Host species	RNA concentration	Number of samples in sequencing pool	Number of paired reads	Number of paired reads after removal of host	Number of contigs >500bp	Number of contigs >500bp of target virus origin
ASCV	G651	Aquaculture Audit	Atlantic salmon	520 ug/ml (added 2.5ug)	4 (17pM/2%phiX)	7928170	373606	211	1
CTV-2	G637	Aquaculture Audit	Atlantic salmon	352 ug/ml (added 2.5ug)	4 (17pM/2%phiX)	8923441	508590	140	3
PTotiV	B5589	Aquaculture Audit	Atlantic salmon	208ug/ml (added 2.5ug)	4 (18.5pM/2% phiX)	8378477	130263	213	0
PNarnaV	G518	Aquaculture Audit	Chinook salmon	812ug/ml (added 2.5 ug)	3 (16pM/2%phiX)	10172852	338528	528	0
PRNAV	B2175	High seas	Chinook salmon	1180ug/ml (added 2.5ug)	4 (17pM/2%phiX)	7158558	30656	43	0

Supplementary table 6 Sequencing statistics for each sequence library. 'Target virus' describes the newly sequenced virus captured from each sequencing run.

Virus	Domain/ ORF	Amino acid alignment length
ASCV	RNA-dependent RNA polymerase	451
CTV-2	ORF1 Polyprotein	1712
PTotiV	RNA-dependent RNA polymerase	103
pNarnaV	RNA-dependent RNA polymerase	111
pRNAV	RNA-dependent RNA polymerase	129

Supplementary table 7 Additional information on the predicted amino acid sequences used to infer phylogenetic relationships.
